# Role of Periapical Diseases in Medication-Related Osteonecrosis of the Jaws

**DOI:** 10.1155/2017/1560175

**Published:** 2017-10-04

**Authors:** Nian Jing Rao, Jing Yi Wang, Ru Qing Yu, Yiu Yan Leung, Li Wu Zheng

**Affiliations:** Discipline of Oral & Maxillofacial Surgery, Faculty of Dentistry, The University of Hong Kong, Pokfulam, Hong Kong

## Abstract

**Objective:**

The present study aimed to investigate the role of periapical diseases in inducing medication-related osteonecrosis of the jaws (MRONJ) using an ovariectomized (OVX) mice model.

**Materials and Methods:**

Twenty C57BL/6N female mice were randomly assigned to two groups. All mice were subjected to bilateral ovariectomy and then treated with oncologic dose of zoledronic acid (ZA) or vehicle for twelve weeks. Eight weeks after commence of drug administration, a pulpal exposure (PE) operation was performed on the first right lower molar to induce periapical periodontitis; the contralateral non-PE tooth was used as control. All animals were sacrificed four weeks after pulpal exposure, and the mandibles were harvested for radiological and histomorphometrical analysis.

**Results:**

Micro computed tomography (*μ*-CT) examination demonstrated that periapical diseases significantly increased alveolar bone resorption, and the resorption was greatly attenuated by ZA treatment. Concurrent ZA therapy significantly increased bone density and histological osteocyte necrosis in the presence of periapical lesions.

**Conclusion:**

ZA treatment reduced bone absorption resulting from periapical disease but increased the risk of developing MRONJ in the ovariectomized mouse model.

## 1. Introduction

Medication-related osteonecrosis of the jaws (MRONJ) is a critical clinical problem which is characterized by the progressive destruction and death of bone tissue of the jaws as a consequence of antiresorptive drug administration. So far the understanding of the mechanism and pathogenesis is still inadequate to justify the most effective treatment of MRONJ. Surgical intervention such as tooth extraction was reported to be a significant triggering factor in inducing MRONJ [1, 2]. However, more recent data proves that tooth extractions are not causative; it is important to realize that severe odontogenic infectious diseases, such as dental caries and periodontal diseases [3], accounted for over 50% of tooth extractions. In addition, oral preventive measures were stated to significantly decrease the incidence of MRONJ, which in turn emphasizes the significance of dental disease in its pathogenesis. This leads to the clinical question of whether the treatment of the diseased condition or the diseased condition itself is the etiological factor of MRONJ.

Clinical studies have revealed potential relevance between uncontrolled periodontitis and MRONJ [4, 5]. This finding was also confirmed by animal experiments including our recent studies [6–8]. Periodontal diseases are anatomically more superficial since the infection is mainly confined to the periodontal pocket and adjacent bone tissue. Periapical disease as a result of dental caries is also among the highest prevalence dental diseases. In contrast to periodontitis, periapical disease may introduce bacteria deeper into the alveolar bone through the root canal systems. It is therefore reasonable to hypothesize that periapical infection may play a role in the development of MRONJ.

The objective of this study was to investigate the role of periapical disease in inducing MRONJ using an ovariectomized (OVX) mouse model.

## 2. Materials and Methods

### 2.1. Animal Care and Surgery

The study was approved by the Committee on the Use of Live Animals for Teaching and Research (CULATR number 3084-13) of The University of Hong Kong. Forty C57BL/6N female mice (body weight 20–25 g, 12 weeks old) were used in the present study. All animals were kept in a dedicated animal holding facility under veterinary supervision in the Laboratory Animal Unit of the Li Ka Shing Faculty of Medicine, The University of Hong Kong. The animals were housed in a 12 : 12 h light-dark circle with 5 mice in a 1144B cage (332.0 × 150.0 × 130.0 mm^3^), which was provided with filtered air at a temperature of 20°C ± 5°C. All mice were allowed free access to water and fed with standard rodent diet (Irradiated, PMI, USA).

Twenty mice were randomly assigned to four groups receiving neither zoledronic acid (ZA) administration nor pulpal exposure (PE) surgery (group A); ZA administration alone without PE surgery (group B); PE surgery alone without ZA administration (group C); or ZA administration and PE surgery (group D). All the mice were subjected to bilateral OVX surgery using the protocol described in our previous study [9]. Six weeks later, vehicle (Veh) or 66 *μ*g/kg of ZA (Zometa® Novartis, Switzerland) solution was administered via intraperitoneally injections, three times per week for 12 weeks. This dose corresponds to the 4 mg/60 kg ZA administered monthly to cancer patients for controlling bone complications, adjusted on a milligram per kilogram basis [6].

To induce periapical diseases, after 8 weeks of ZA/Veh administration, a pulpal exposure (PE) was created on their first left lower molar (M1) on all animals under general anesthesia. The coronal ceiling of the molar was removed using a 1/4 spherical bur and the drilling was proceeded to a depth of the diameter of the bur to avoid perforation of the furcal floor. After exposure, the tooth was left open to the oral cavity for four weeks to contaminate the root canals with the animals' own oral flora [10]. Before and after the operation, analgesic and anesthetic were administrated.

All mice were sacrificed 4 weeks after the pulpal exposure. The entire mandible was harvested and the overlying soft tissues were removed carefully. All the samples were then fixed in 10% neutral buffered formalin solution for further examinations.

### 2.2. Clinical Examination

The whole oral cavity was thoroughly examined for any signs of redness, ulceration, and the presence of necrotic bone.

### 2.3. *μ*-CT Examination

All specimens were scanned using a micro tomography (*μ* CT) system (SkyScan-1172 X-ray micro tomography, SkyScan, Kontich, Belgium) following the manufacture's instruction (voltage = 80 kV, current = 100 *μ*A, and exposure = 3993 ms). Three-dimensional images were accessed at a resolution of 8 *μ*m/pixel and the images were reconstructed using NReconserver 1.6.7 software (SkyScan, Kontich, Belgium) and analyzed with CTAn program (CTAn 1.12.0, SkyScan).

#### 2.3.1. Trabecular Microstructure Changes

To assess the trabecular microstructure, alveolar bone inside the interradicular septum of M1 was determined as the region of interest (ROI). A set of parameters including bone mineral density (BMD), trabecular thickness (Tb.Th), bone volume/tissue volume (BV/TV), trabecular separation (Tb.Sp), and trabecular number (Tb.N) were assessed [11, 12].

#### 2.3.2. Alveolar Bone Resorption

In order to assess the effectiveness of PE-induced periapical periodontitis and the effect of ZA on alveolar bone resorption, periapical and periodontal bone loss were measured. Periapical bone loss was measured from the root apex to the periapical alveolar bone, which was measured at the mesial root of the M1. The amount of periodontal bone loss was assessed by the width of the periodontal ligament (PDL) space at the furcation area of the M1. The PDL width was measured at the middle of the mesial surface of the distal root, the tip of the furcation, and the middle of the distal surface of the mesial root [12].

### 2.4. Histological Examination

After CT scanning, all samples were decalcified with 14.5% EDTA (PH 7.2) at 25°C for 2 months. After being dehydrated with graded ethanol (70%, 95%, and 100%), the specimens were cut from the midline and embedded separately in paraffin. To further assess the micro osteonecrosis in the alveolar bone, the embedded specimens were sectioned in the buccal-lingual direction into 5 *μ*m thickness slices and subjected to hematoxylin and eosin (HE) staining.

Histological osteonecrosis was determined as five contiguous empty osteocytic lacunae present in trabecular bone together with the loss of osteocytes [8]. For quantitative analysis, the number of nonviable osteocytes was counted in the three randomly selected high power fields and counted manually using the protocol described in our previous study [8].

### 2.5. Statistical Analysis

The main effects of ZA administration (ZA versus Veh) and periapical lesions caused by pulpal exposure (PE versus non-PE) and their interactions were tested by two-way ANOVA. If significant, further assessments of independent *t*-test were performed to determine the difference among or between groups. *P* values < 0.05 were considered statistically significant. All analysis was conducted with IBM SPSS statistics software (version 23.0, IBM Crop, Armonk, NY, USA).

## 3. Results

### 3.1. Clinical Examination

All animals (20/20) completed the experiment uneventfully. Exposed coronal cavities were present on the drilled teeth. None of the animals in both ZA and Veh control groups exhibited clinical signs of infection. No oral mucosal lesion or exposed bone was observed.

### 3.2. *μ* CT Analysis

#### 3.2.1. BMD and Bone Microstructure Measurement

**Figure 1:**
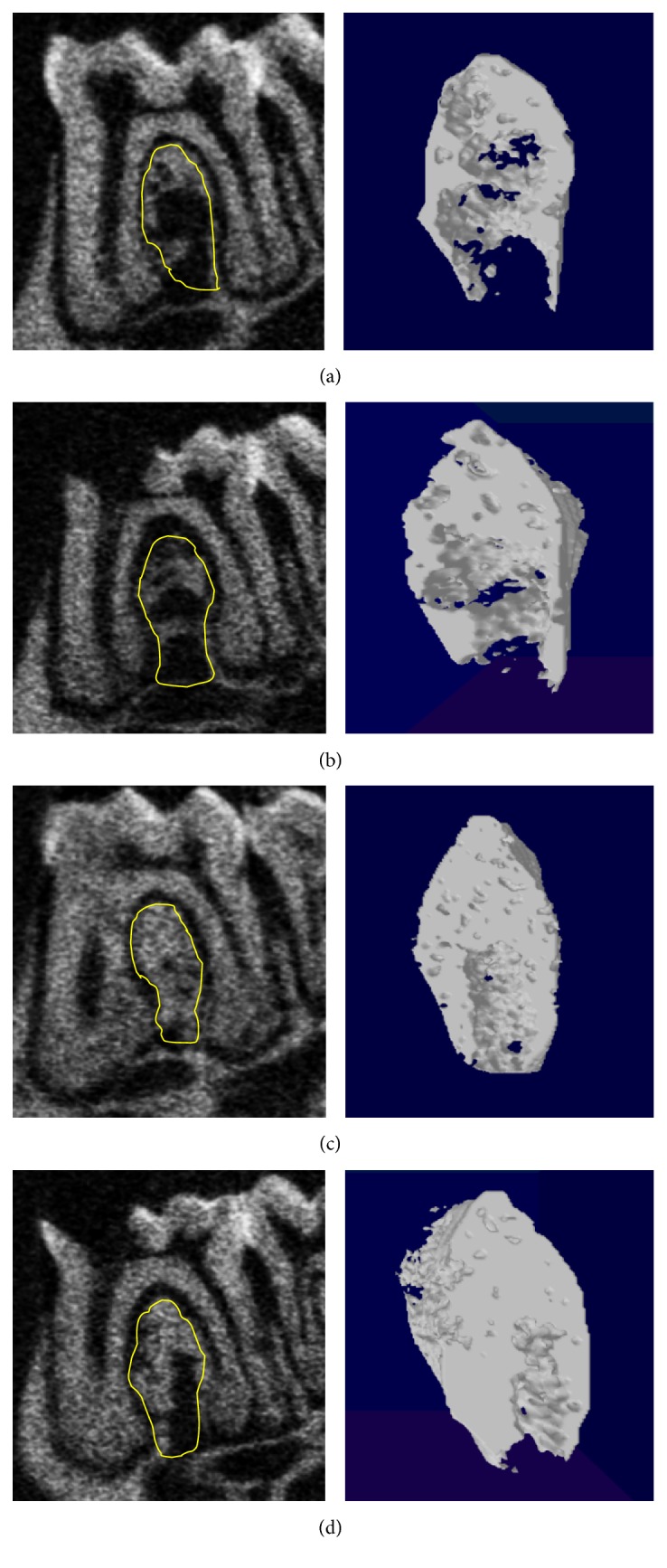
Trabecular bone inside the yellow circle is selected as ROI for micro-CT analysis. (a) Non-PE tooth in vehicle group. (b) PE tooth in vehicle group. (c) Non-PE tooth in ZA group. (d) PE tooth in ZA group. Images on the right side are the 3D images of the selected area. ZA animals demonstrate increased density and more abundant bone mass.

Significant interaction was found between the effects of ZA administration and pulpal exposure on BMD (*P* = 0.016). Simple main effects analysis showed that the BMD was significantly lower in the PE teeth than in non-PE teeth when administered with vehicle (*P* < 0.005), but there were no differences between PE and non-PE teeth in the ZA group (*P* = 0.538). Mice in the ZA group had a significantly increased BMD compared to the control vehicle groups with or without pulpal exposure (*P* < 0.005) (Table 1) (Figure 1).

The effects of ZA and PE on microarchitecture of the intraradicular trabecular bone were examined by two-way ANOVA after being tested for normality distribution and homogeneity of variance. There was no significant interaction between the effects of ZA administration and periapical diseases on alveolar bone microarchitecture. Pulpal exposure did not cause any significant change on the bone microstructure either. However, ZA groups showed significant increase of BV/TV and Tb.Th and decrease in Tb.N (Table 1).

#### 3.2.2. Alveolar Bone Loss

There was a statistical significant interaction between the effects of ZA and PE on the width of periodontal ligament space (PDL). Simple main effects analysis showed that the mean width of PDL was significantly smaller in ZA groups than in Veh groups when the teeth were drilled (*P* < 0.0005), but no substantial difference was found between ZA and non-ZA animals in the non-PE group (*P* = 0.868). PE significantly increased the periapical bone resorption and the width of PDL in both ZA and vehicle groups (*P* < 0.05).

### 3.3. Histological Examination

**Figure 2:**
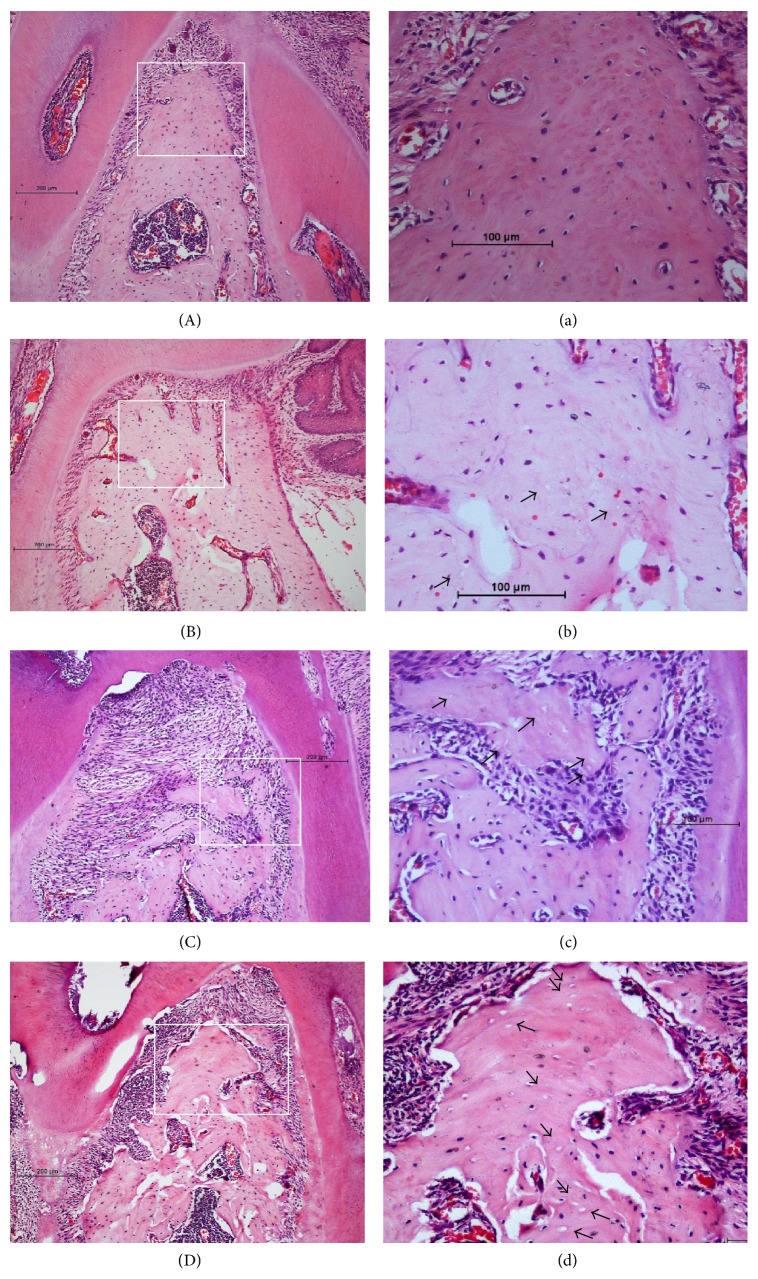
Histological images of the alveolar bone (HE staining). (A) Non-PE tooth in vehicle group. (B) Drilled tooth in vehicle group. (C) Nondrilled tooth in ZA group. (D) Drilled tooth in ZA group. a, b, c, and d are magnified views of the square area from A to D. Empty osteocytic lacunae present in the alveolar bone (arrows).

Histological examination showed healthy dental pulp tissues were observed in non-PE teeth, and PE teeth in both groups (ZA and Veh) presented the signs of pulpitis, pulp necrosis, alveolar bone resorption, and inflammatory infiltration around the drilled teeth (Figure 2). The results confirmed that pulpal exposure was sufficient for stimulating pulpal and periapical inflammatory reactions in mice.

**Figure 3:**
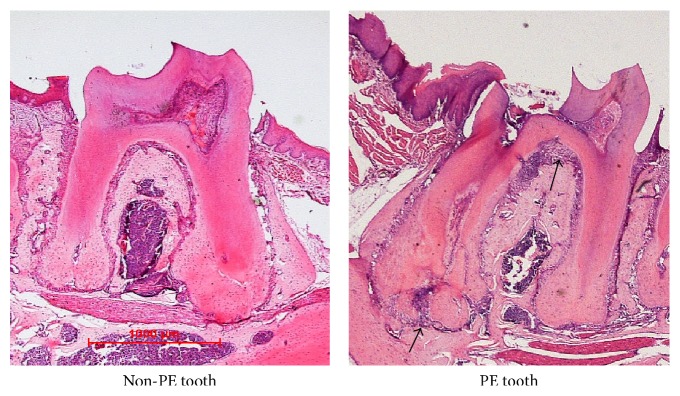
Histological images of the pulp exposed (PE) and nonexposed (non-PE) molars (HE staining). Healthy pulp tissue is observed in non-PE tooth, while signs of pulpitis, pulp necrosis, periodontal and periapical bone loss, and inflammatory infiltration (black arrows) are observed in the pulpal exposed tooth.

Both ZA treatment (*P* = 0.009) and pulpal exposure (*P* < 0.0005) had significant effects on alveolar bone micronecrosis (Figure 3). There was no statistically significant interaction between the effects of ZA and PE (*P* = 0.921). However combined ZA and PE did result in an increased number of nonviable osteocytes (Table 2).

## 4. Discussion

Rodents have been consistently the choice of animal model for many human diseases. In addition to their genetic similarity with human, their completed genome mapping could further facilitate genetic engineering exploration and allow researchers to study molecular events due to the availability of probes. However, rodents do not normally show coupled remodeling in the cortical bone. This characteristic poses a limitation as an experimental model for MRONJ, as the pathogenesis is thought to be associated with aberrant intracortical remodeling [13, 14]. Studies including our recent experiments have demonstrated that active intracortical remodeling can be stimulated by bilateral ovariectomy on rodents [9, 15, 16]. Therefore an ovariectomized mouse model was used in the present study.

On the basis of the equivalent therapeutic dose (4 mg/60 kg monthly) used for cancer patients to control bone metastases, 66 mg/kg of ZA or vehicle was injected intraperitoneally three times per week for 8 weeks prior to pulpal exposure and another 4 weeks afterward. This is to ensure enough time for bisphosphonates (Bps) drugs to deposit and accumulate in the bone before any interventions. Accordingly, a total of 36 oncologic doses of ZA which is equivalent to three years of ZA therapy in human were given to the animals in this study.

Tooth extraction and traumatic oral procedures were believed to be important etiologic factors for the onset of MRONJ [13, 17–21]. Besides trauma, however, infection may also play a critical role in the development of ONJ induced by antiresorptive drugs including Bps. Of note, most tooth extractions were performed as a result of severe odontogenic infectious diseases. The causal relationships between infection, tooth extraction, and MRONJ are still largely unknown.

In the present study, periapical lesions were induced by tooth coronal opening to create a pulpal exposure [10, 22–25]. Radiographically, PE teeth displayed significantly increased periodontal ligament space and periapical bone resorption, whereas the undrilled non-PE teeth did not show any obvious signs of alveolar bone resorption. Pulp opening resulted in distinct periapical bone resorption and intensive periodontal bone destruction. ZA did not show a significant effect in decreasing periapical bone loss in the presence of periapical diseases. However, it substantially reduced periodontal bone loss. With regard to bone structure, bone loss is usually associated with the degradation of skeletal architecture, which is characterized by a reduction in the number of trabeculae, an increase in intertrabecular distances, and a disruption of the connectivity of the trabecular meshwork. Despite the significant increased bone resorption, periapical diseases did not cause any remarkable changes on alveolar bone architecture in animals received ZA. Moreover, ZA administration significantly increased BV/TV and Tb.Th and decreased Tb.N in both PE and non-PE teeth.

An interaction between Bps and periapical diseases on changes of intraradicular bone BMD was also found in this study. While significant BMD reduction was stimulated by periapical inflammation, ZA appeared to offset this effect, which is in accordance with its biological bone protection description [26, 27]. Although potent nitrogen-containing bisphosphonate (N-Bps) has been exhibiting preservation or improvement of bone strength constantly in numerous studies using different animal models with Bps given at various doses and time intervals, there have been concerns that the inhibition in bone turnover by Bps may compromise bone integrity. Excessive inhibition may result in increased bone fragility [28]. In our study, greater BMD, bone volume, bone thickness, and fewer trabeculae were observed consistently in ZA treated animals, which indicates increased density and mechanical strength.

In addition to the abnormal radiographic findings, histological osteonecrosis was observed in animals that received ZA and PE. Both ZA and PE treatments showed significant effects in increasing micronecrosis of the alveolar bone; however, the characteristic exposed bone lesion did not appear in any animals in this study. One possible explanation for the absence of exposed bone could be the nontraumatic intraoral interventions we used. Unlike most ONJ studies where tooth extractions as well as other aggressive trauma manipulations were performed, ZA took effect in the absence of mucosa damage in the present study. Another factor may be the absence of bacteria in the rearing condition of animals. There were probably very few bacteria in the oral cavity of these mice due to the relatively germ-free conditions they were kept [29]. In addition, given the inherent metabolic differences between species, the clinical diagnostic criteria of MRONJ in human might not be directly applicable to animals [30]. Moreover, the average treatment duration of 20 months is usually required for MRONJ occurrence under the therapy of high potent N-Bps such as ZA (Palaska et al., 2009). However, most of the animal studies had much shorter time than that to evaluate MRONJ lesions due to the limitation of the rodents' lifespan [14].

The short time period after intervention (4 weeks) might also contribute to the absence of clinically bone exposure in this study. Even though the results of this study conformed to the clinical criteria of Stage 0 MRONJ, which applied to those cases where the bone necrosis precedes mucosal retraction and bone exposure [31, 32], the characteristic clinical manifestations of MRONJ could not be expected, and also the conditions to meet the criteria stages 1–3 of MRONJ were excluded as they require at least 8 weeks of bone exposure. So stage 0 was the maximum effect to be expected in this setting.

Another limitation of experimental methods in this study is that pulpal exposure might facilitate pulpal infection but not necessarily the same as pulpal infection. This can certainly influence the results and also the time till occurrence of clinically manifest MRONJ.

Long duration of ZA administration was observed to substantially increase the death of osteocytes in this study. Osteocytes are the most abundant bone cells in mature bone tissue and their death and replacement are the results of natural process of metabolism. However, due to the antiresorptive effect of Bps, nonviable osteocytes degradation could diminish and gradually accumulate [33]. Gradually, the accumulation of nonviable osteocytes may lead to the necrosis of bone. In addition, there is hypothesis indicated that Bps exert a direct toxic effect on osteocytes and cause their death. It has been reported that fluorescently labeled systemically administered Bps were found embedded around the lacunae. It could be expected that the exposure of osteocytes to high concentrations of potent N-Bps over a period of time could impair their viability [34].

Periapical infection and inflammation also demonstrated remarkable accelerating influence on histological necrosis of the bone with or without the presence of ZA. Although there was no significant interaction between the action of ZA and periapical diseases on osteonecrosis, our results showed that periapical infection coupled with N-Bps administration increased the number of nonviable osteocytes. In the present study, significant bone resorption was induced followed by the pulp exposure intervention. Bps are known to bind to bone at neutral pH and are discharged from bone in an acidic environment. During bone resorption, the acid pH in the resorption lacunae increases the release of Bps from hydroxyapatite. Thus, the resulting infection followed by pulp exposure can induce local tissue pH reduction and subsequent increase in Bps release. In addition, a decrease in pH results in protonation activation of the nitrogen-containing groups, thereby increasing the conversion of the derivatives to the potential toxicity level, which in turn may lead to cascading of the pathways accumulated in MRONJ [35]. To date, this unique feature may link infections to the pathogenesis of MRONJ. In humans, local acidic milieus are not uncommon in infections and wound healing after surgical procedures. Similarly, the jawbones are often exposed to odontogenic infectious diseases and surgical procedures.

Although it is still unclear whether nontraumatic odontogenic lesions and a period of ZA therapy are sufficient to trigger a MRONJ lesion, this study formed the basis to further investigate the biochemical and histological factors of the development of MRONJ.

## 5. Conclusions

Both periapical disease and bisphosphonate administration could increase the death of osteocytes. ZA injection reduced bone absorption resulting from the periapical disease but increased the risk of developing MRONJ in this ovariectomized mouse model.

## Figures and Tables

**Table 1:** B1MD and bone microstructure measurement in ZA and vehicle groups.

	ZA		Vehicle	
	PE	Non-PE	PE	Non-PE
BMD (g/cm^3^)	.7228/.1022	.7457/.0825	.3760/.0621	.5312/.0779
BV/TV (%)	79.0763/13.4511	77.3756/6.8013	61.1034/7.2136	61.2206/7.6028
Tb.Th (mm)	.0945/.0106	.0917/.0083	.0858/.0066	.0868/.0079
Tb.Sp (mm)	.5980/.0227	.0634/.0128	.0644/.0115	.0758/.0072
Tb.N (1/mm)	18.65/9.922	20.90/9.527	35.60/18.155	40.20/14.793

**Table 2:** Number of empty lacunae of the nonviable osteocytes (per mm^3^).

Group	Description	Empty lacunae (1/mm^3^)	SD
A	ZA(−)*∗*PE(−)	19.0464	13.8206
B	ZA(+)*∗*PE(−)	34.6660	17.7273
C	ZA(−)*∗*PE(+)	51.4550	14.3618
D	ZA(+)*∗*PE(+)	65.9800	21.9675
